# Association of dyslipidemia, hypertension and overweight/obesity with work shift and duration of employment among police officers in a small town in Northeastern Brazil

**DOI:** 10.5327/Z1679443520190401

**Published:** 2019-12-01

**Authors:** Cícero Adilson Coelho da-Silva, Alexsandra Laurindo Leite, Jéssica Alves Moreira, Dandara Dias Cavalcante Abreu, Pierri Emanoel de Abreu Oliveira, Daniella Pires Nunes, Maria Iranilda Silva Magalhães, José Bruno Nunes Ferreira Silva

**Affiliations:** 1 Department of Clinical Testing, Biomedicine undergraduate course, Faculdade Santa Maria - Cajazeiras (PB), Brazil. Faculdade Santa Maria Department of Clinical Testing Biomedicine undergraduate course Faculdade Santa Maria Brazil; 2 Undergraduate nursing course, Universidade Federal do Tocantins - Palmas (TO), Brazil. Universidade Federal do Tocantins Undergraduate nursing course Universidade Federal do Tocantins Brazil; 3 Undergraduate medical course, Universidade Federal do Tocantins - Palmas (TO), Brazil. Universidade Federal do Tocantins Undergraduate medical course Universidade Federal do Tocantins Brazil

**Keywords:** cardiovascular diseases, dyslipidemias, hypertension, obesity, shift work schedule

## Abstract

**Background:**

Metabolic syndrome and chronic diseases have impact on the job performance of police officers.

**Objective:**

To investigate the association of overweight/obesity and cardiovascular risk factors with work shift and duration of employment among police officers.

**Methods:**

Cross-sectional study with 102 police officers in Cajazeiras, Paraíba, Brazil, in which we analyzed sociodemographic data, occupational characteristics, body mass index (BMI), lipid profile, personal history of disease and lifestyle. Statistical analysis included the chi-square and Fisher’s exact test.

**Results:**

Overweight/obesity was found among most participants (83.3%). Hypertriglyceridemia (49.1%), low HDL-C (56.9%) and high LDL-C (46.1%) levels were associated with abnormal BMI (p<0.05). Hypertension was the main disease reported by overweight/obese participants (28.2%) (p=0.01). Job position, lifestyle and work shift were not associated with any of the analyzed variables, however, abnormal BMI, dyslipidemia, and hypertension were frequent among the participants with 6-10 or more than 10 years in the job (p<0.05).

**Conclusion:**

Part of the participants with at least 6 years in the job exhibited overweight/obesity in association with dyslipidemia and hypertension. We recommend prevention and therapeutic strategies to protect officers from chronic diseases or attenuate their long-term complications. Additional prospective studies are needed to confirm the associations we found, mainly between duration of employment and occupational diseases.

## RESUMO |

**Introdução:**

A síndrome metabólica e diversas doenças crônicas vêm impactando o trabalho de policiais militares.

**Objetivos:**

Avaliar a relacão entre sobrepeso/obesidade e fatores de risco cardiovascular associados ao turno e ao tempo de serviço entre policiais.

**Métodos:**

Estudo transversal com 102 policiais militares da cidade de Cajazeiras, Paraíba. Foram analisadas características sociodemográficas, ocupacionais, índice de massa corporal (IMC), perfil lipídico, comorbidades e estilo de vida. Os testes χ^2^ e exato de Fisher foram utilizados na análise dos grupos.

**Resultados:**

Sobrepeso/obesidade foi encontrado em 83,3% dos policiais. Hipertrigliceridemia (49,1%), baixas concentrações de HDL-C (56,9%) e LDL-C sérico elevado (46,1%) apresentaram associação com alterações do IMC (p<0,05). Hipertensão arterial foi a principal doença relatada pelos participantes com sobrepeso/obesidade (28,2%) (p=0,01). Função desempenhada, estilo de vida e turno de trabalho não apresentaram associação com as variáveis analisadas. No entanto, alterações no IMC, dislipidemia e hipertensão foram frequentes entre os participantes com 6 a 10 ou mais de 10 anos de serviço (p<0,05).

**Conclusão:**

Parte dos participantes com um mínimo de 6 anos de tempo de serviço apresentou sobrepeso/obesidade associado a dislipidemia e hipertensão arterial. Recomenda-se a implementação de estratégias preventivas e terapêuticas para proteger os agentes policiais de doenças crônicas ou atenuar suas complicações no longo prazo. Estudos prospectivos são necessários para confirmar as associações identificadas no presente estudo, principalmente entre o tempo de serviço e doenças ocupacionais.

## INTRODUCTION

Police officers are civil servants charged of citizen protection, peace preservation and promotion of violence reduction. Pre-employment examination of police officers in Brazil includes intellectual, cognitive and personality assessment, physical examination, laboratory tests and social evaluation. To perform their tasks, no matter whether administrative or operational, police officers need to maintain a high level of physical, mental and behavioral health all along their career. However, several studies found association between work and chronic diseases, behavioral changes and metabolic syndrome (MetS) among police officers and military personnel^1^^,^^2^.

Obesity is a heterogeneous chronic disease that involves accumulation of body fat. Body mass index (BMI), a measure of weight relative to height, is used to identify overweight and obese people; cut-off points are the same for men and women regardless their age. Chronic conditions such as cardiovascular disease, dyslipidemia, hypertension, insulin resistance and type 2 diabetes mellitus are associated with changes in BMI^3^. Physical or psychological symptoms ­and/­or functional limitations related to obesity may impair the quality of life of the affected individuals in their work and social lives.

Studies performed in Brazil sought to establish how body fat accumulation influences work performance and occurrence of chronic diseases among police officers. High body fat percentages are associated with poor quality of life and impaired reaction time in the job^4^^,^^5^. Overweight and obesity impair physical activity and increase cardiovascular risk among highway and urban police officers^6^^,^^7^. Dietary fat intake above the recommended levels correlates with increased abdominal fat among this population of workers^8^.

Police officers usually have long working hours, eventually of 24 uninterrupted hours. Shift work has been implicated as a risk factor for cardiovascular disease, MetS, increased waist circumference and abnormal BMI^9^^,^^10^ and disrupts the circadian rhythm^11^. To the best of our knowledge, no previous study investigated associations between BMI, lipid parameters, hypertension, shift work and duration of employment among urban police officers in small Brazilian towns. The hypothesis underlying the present study was that shift work and duration of employment have influence on overweight/obesity and contribute to the occurrence of occupational diseases among police officers. Therefore, the aim of the present study was to investigate the association of overweight/obesity and cardiovascular risk factors with shift work and duration of employment among police officers in Cajazeiras, Paraíba, Brazil. In addition, we also analyzed the participants’ personal medical history.

## METHODS

### STUDY DESIGN

The present cross-sectional analytical study is based on data collected through administration of a questionnaire to police officers in Cajazeiras. We also measured the participants’ body weight and height and collected blood samples. Cajazeiras is located in the state of Paraíba in the Northeast region of Brazil, and had a population of 62,187 in 2017^12^. Participants were recruited from the 6th Military Police Battalion and the 5th Traffic Police Company, which included a total of 215 officers in 2017. We interviewed 102 officers from the administrative, patrolling, special forces and traffic departments, who signed an informed consent form as required by the National Health Council Resolution no. 466/12.

The study was approved by the research ethics committee of Santa Maria School, ruling no. 2.328.175. Data collection was performed in November and December 2017. Anonymity and confidentiality were ensured; the interviewees were identified through numerical codes. All the gathered information was exclusively used for study purposes.

### DATA COLLECTION

The questionnaire administered to the participants investigated sociodemographic characteristics (age, sex, educational level, marital status, daily working hours, position, duration of employment and sleep time), alcohol consumption, smoking, dietary habits and physical activity. The participants were also inquired as to their medical history, particularly cardiovascular disease, diabetes mellitus, food intolerances/allergies, gastric ulcer, gastritis/stomachache, low blood pressure, low back pain, obesity, psychiatric disorders and hypertension acquired after entering the job.

### OBESITY AND BIOCHEMICAL PARAMETERS

We measured the participants’ body weight and calculated BMI as body mass (kg) divided by height squared (m^2^). The participants were categorized as with normal weight (18.5 to <25 kg/m^2^), overweight (25-29.9 kg/­m^2^) or obesity (≥30 kg/m^2^)^13^. Biochemical parameters were evaluated as per the latest update of the V Guidelines on Dyslipidemias and Atherosclerosis Prevention formulated by the Brazilian Society of Cardiology in 2017^14^ in accordance with European guidelines for management of dyslipidemias^15^. The participants were required to fast 12 hours before blood sample collection. The samples were centrifuged at 3500 rpm for 15 minutes and the serum was used to measure total cholesterol (TC), triglycerides (TG), high-density lipoprotein-cholesterol (HDL-C) and low-density lipoprotein-cholesterol (LDL-C) with LABTEST kits (Labtest Diagnóstica SA, Minas Gerais, Brazil) and LABMAX 560 analyzer. TC <190 mg/dL, TG <150 mg/­dL, HDL-C ≥40 mg/dL and LDL-C <130 mg/­dL were considered acceptable levels as per the latest Brazilian guidelines^14^.

## STATISTICAL ANALYSIS

We performed descriptive statistics to characterize the study sample. Age and biochemical parameters were expressed as mean±standard deviation; categorical variables were reported as percentages. The participants were divided in two groups: normal weight and overweight/obesity. Differences in proportions between the groups and associations among categorical variables were subjected to the χ^2^ or Fisher’s exact tests. We also analyzed the proportion of cases of hypertension in both groups. The data were analyzed using IBM Statistical Package for the Social Sciences (SPSS) for Windows, version 25.0 (IBM Corp., Armonk, NY).

## RESULTS

A total of 102 police officers responded the questionnaire, being 8 women and 94 men, with average age 39.10±7.48 years old. Most participants had completed secondary or higher education and had more than 10 years in the job. About 75% of the sample worked 24-hour shifts, 33% were assigned to patrolling, 28.4% to administrative tasks, 26.2% to special operations and 11.8% to traffic control and enforcement (Table 1). Next, we analyzed the participants’ lifestyle and found that 96.1% of the sample were non-smokers, while 60.8% reported to consume alcohol. Most participants performed physical activity one to three times per week, 37.3% did not exercise outside the workplace and 75.5% reported to sleep 6 or more hours/night when off duty (Table 1).

**Table 1.** Sociodemographic and occupational characteristics and lipid profile of police officers in Cajazeiras, Paraíba, Brazil, distributed according to by BMI, 2017 (n=102).

	Total (N=102) (%)	Normal weight (N=17) (%)	Overweight/Obesity (N=85) (%)	p value
Sex*				
Female	8 (7.8)	3 (37.5)	5 (62.5)	0.127
Male	94 (92.2)	14 (14.9)	80 (85.1)	
Age* (years)				
18-29	5 (4.9)	2 (11.8)	3 (3.53)	0.225
30-39	61 (59.8)	11 (64.7)	50 (58.82)	
≥40	36 (35.3)	4 (23.5)	32 (37.65)	
Marital Status				
Married	83 (81.4)	11 (64.7)	72 (84.7)	0.083
Single	19 (18.6)	06 (35.3)	13 (15.3)	
Educational level*				
Elementary school	16 (15.7)	2 (11.8)	14 (16.5)	0.850
Secondary school	38 (37.3)	8 (47.1)	30 (35.5)	
Incomplete/ongoing higher education	11 (10.8)	1 (5.9)	10 (11.8)	
Complete higher education	37 (36.3)	6 (35.3)	31 (36.5)	
Daily working hours*				
6	14 (13.7)	1 (7.1)	13 (92.9)	0.053
12 or 18	9 (8.8)	4 (44.4)	5 (55.6)	
24	79 (77.5)	12 (15.2)	67 (84.8)	
Years in the job*				
0-5	4 (3.9)	3 (75)	1 (25)	0.010
6-10	42 (41.2)	8 (19)	34 (81)	
> 10	56 (54.9)	6 (10.7)	50 (89.3)	
Dietary intake*				
Breakfast				
Before work	76 (74.5)	14 (18.4)	62 (81.6)	0.587
At work	18 (17.65)	03 (16.7)	15 (83.3)	
None	8 (7.85)	-	8 (100)	
Type of food (yes)				
Candy*	39 (38.2)	3 (17.6)	36 (42.4)	0.062
Canned food	20 (19.6)	6 (35.3)	14 (16.5)	0.095
Coffee	72 (70.6)	9 (52.9)	63 (74.1)	0.080
Fast-food	45 (44.1)	10 (58.8)	35 (41.2)	0.181
Soft drinks	32 (31.4)	6 (35.3)	26 (30.7)	0.703
Smoking*				
Yes	4 (3.9)	0	4 (4.7)	1.000
Alcohol consumption				
Yes	62 (60.8)	11 (64.7)	51 (60)	0.717
Physical activity/week*				
1 to 3 times	42 (41.18)	7 (41.2)	35 (41.2)	1.000
>3 times	22 (21.57)	4 (23.5)	18 (21.2)	
None	38 (37.25)	6 (35.3)	32 (37.6)	
Sleep time/day (hours)*				
<6	25 (24.5)	3 (17.6)	22 (25.9)	0.554
≥6	77 (75.5)	14 (82.4)	63 (74.1)	
Lipid profile [n(mean±df; %)]				
TC				
Acceptable	56 (157.84±23.28; 54.9)	10 (9.8)	46 (45.1)	0.722
High	46 (212.13±20.20; 45.1)	7 (6.9)	39 (38.2)	
HDL-C				
Acceptable	37 (48.32±5.80; 36.3)	10 (9.8)	27 (26.5)	0.034
Low	65 (31.98±5.41; 63.7)	7 (6.8)	58 (56.9)	
LDL-C				
Acceptable	51 (80.68±21.15; 50)	13 (12.7)	38 (37.3)	0.031
High	51 (131.65±22.57; 50)	4 (3.9)	47 (46.1)	
TG				
Acceptable	48 (106.94±31.16; 47)	13 (12.7)	35 (34.3)	0.015
High	54 (266.13±125.14; 53)	4 (3.9)	50 (49.1)	

*Fisher or χ^2^ test; TC: total cholesterol; HDL-C: high-density lipoprotein-cholesterol; LDL-C: low-density lipoprotein-cholesterol; TG: triglycerides.

Sixty-five participants exhibited overweight and 20 obesity and were analyzed together as a single group (Table 1). Nineteen participants had class I obesity and one class II obesity. There was no statistically significant difference between the participants with normal weight and overweight/obesity in regard to age, educational level or marital status. The frequency of overweight/obesity was higher among the participants who worked 6- or 24-hour shifts (p=0.053). The proportion of abnormal BMI was higher among the participants with at least 6 years in the job (p=0.010). We did not find any association between overweight/obesity, smoking status, alcohol consumption or dietary intake (Table 1).

The relationship between BMI and lipid profile is described in Table 1. The overweight/obese group exhibited lower HDL-C (p=0.034), higher LDL-C (p=0.031) and higher TG (p=0.015). We did not find any association between TC and BMI. Thirty-eight participants with 6-10 years and 53 participants with more than 10 years in the job had dyslipidemia (p=0.002). On analysis of the participants’ medical history after entering the job we found a larger proportion of cases of hypertension in the overweight/obesity group (p=0.01) (Table 2).

**Table 2.** Proportion of participants with normal weight or overweight/obesity according to personal medical history, Cajazeiras, Paraíba, Brazil, 2017 (n=102)*.

	Normal weight (N=17) (%)	Overweight/Obesity (N=85) (%)	p value
Personal medical history			
Cancer	0.0	1.2	1
Cardiovascular disease	0.0	1.2	1
Food intolerance/allergy	11.8	4.7	0.261
Gastritis/stomachache	33.3	36.5	0.801
Hypertension	0.0	28.2	0.01
Low back pain	17.6	14.1	0.712
Psychiatric disorders	0.0	1.2	1
Type 2 diabetes	0.0	2.4	1

*Fisher’s test.

Table 3 describes the proportion of participants in the overweight/obesity group with hypertension according to job position, duration of employment, working hours and lipid profile. Administrative and special forces officers were the most likely to exhibit hypertension (p=0.201). The proportion of participants with hypertension was higher among those who worked 24-hour shifts, but this result was not statistically significant. Most of the participants with high blood pressure had at least 6 years in the job (p=0.022). We found high TG levels in 83.3% of the participants with hypertension (p=0.001), 62.5% of the latter had high TG combined with low HDL-C levels (p=0.019) and did not exercise outside the workplace (p=0.014).

**Table 3.** Proportion of overweight/obese participants with hypertension according to occupational characteristics, lipid profile and physical activity, Cajazeiras, Paraíba, Brazil, 2017 (n=24)*.

	Hypertension (%)	p value
Job position		
Administrative	41.7	0.201
Patrolling	12.5	
Special operations	37.5	
Traffic	8.3	
Daily working hours		
6	16.7	0.915
12 or 18	8.3	
24	75	
Years in the job		
0-5	0	0.022
6-10	20.8	
>10	79.2	
TG levels		
Acceptable	16.7	0.001
High	83.3	
Low HDL + high TG levels		
Yes	62.5	0.019
No	37.5	
Physical activity (times/week)		
1 to 3	29.2	0.014
>3	8.3	
None	62.5	

*Fisher’s test; TG: triglycerides; HDL: high-density lipoprotein.

## DISCUSSION

The results indicate that 83.3% of the participants exhibited overweight/obesity. In other studies this rate varied from 55.15 to 73.87% among police officers in the Central-West, Northeast and South regions of Brazil^8^^,^^16^^,^^17^. ­Overweight/­obesity was associated with low HDL-C levels. Low plasma HDL-C levels may occur independently from hypertriglyceridemia, be secondary to high levels of inflammatory cytokines, and are associated with excess fat^18^^,^^19^. Our data show that overweight/obese police officers exhibited typical dyslipidemia-hypertriglyceridemia and high LDL-C levels.

The rate of overweight/obesity was higher among the participants who worked 6- or 24-hour shifts. Also a previous study found positive correlation between BMI and duration of employment among special forces and traffic police officers^20^. Thirteen the 14 overweight/obese officers who worked 6-hour shifts had at least 6 years in the job. The frequency of abnormal BMI was higher among the participants who worked 24-hour shifts. Long working hours, inadequate diet, changes in the circadian rhythm and stress had been associated with abnormal BMI among police officers^10^. In the present study, 66 of the 77 participants with ­overweight/­obesity who worked 24-hour shifts had at least 6 years in the job (p=0.018). Recently, Joo et al.^21^ found a relationship between night work and dyslipidemia among male workers. In the present study, abnormal LDL-C was more common among shift workers^22^. We also found association between duration of employment and dyslipidemia.

Hypertension only occurred among the ­overweight/­obese officers. This result corroborates those reported by Brown et al.^23^ and Sarno and Monteiro^24^ which indicate strong association between abnormal BMI and occurrence of hypertension and dyslipidemia among adults. Wenzel et al.^1^ found that the prevalence of hypertension was three times higher among obese air force personnel in Brazil; the rate of excess weight was twice higher than that of normal weight. Other studies performed in Brazil reported hypertension rates of 5.3% to 55.76% among police officers^17^^,^^18^. Although the influence of shift work on hypertension has been already demonstrated^25^ it remains controversial because night work does not increase the blood pressure^26^. While we did not find any correlation among these variables, the proportion of participants with high TG or low HDL-C + high TG was higher among the participants with hypertension. Dyslipidemia is considered a cardiovascular risk factor and a predictor of hypertensive crisis^27^. The participants with hypertension did not perform physical activity outside the workplace, which might impair their quality of life, since lack of exercise may cause chronic diseases. In turn, several studies reported beneficial effects of physical activity on hypertension^28^.

The present study has some limitations, to begin with its cross-sectional design which precludes inferring causal relationships. By contrast, we focused on BMI, hypertension and dyslipidemia, because they are frequently considered to be cardiovascular risk factors and thus demand a long-term approach. In addition, the mechanisms underlying dyslipidemia in obesity^29^ and hypertension^27^, as well as of obesity-induced hypertension^30^, have been well established. Second, the data were obtained from a small number of officers at two police departments in Cajazeiras. Although most studies performed in Brazil focused on police officers in major urban centers across the country, and we selected a small rural town, the results were similar. Third, we did not administer a previously validated questionnaire to assess physical and behavioral disorders. Nevertheless, we believe that self-reported comorbidities and the personal and family medical history are clinically relevant in the assessment of any disease.

## CONCLUSION

To summarize, the results of the present study evidenced a high prevalence of overweight/obesity among police officers in Cajazeiras. Since overweight/obesity and abnormal lipid profile and blood pressure are strongly correlated with cardiovascular disease, these findings may serve to trigger changes in the participants’ lifestyle to thus minimize long-term complications. Dyslipidemia and hypertension were associated with at least 6 years in the job. Preventive, therapeutic and behavioral strategies to protect police officers from chronic diseases or minimize their long-term complications are beneficial not only for these workers, but also for the overall population. Prospective studies are needed to establish whether duration of employment has or not strong association with abnormal BMI, dyslipidemia and hypertension among workers.
